# Brief parenting intervention (Triple P) for families of children with eczema: a randomized controlled trial

**DOI:** 10.1093/jpepsy/jsae023

**Published:** 2024-04-10

**Authors:** Amy E Mitchell, Alina Morawska, Emily Casey, Elana Forbes, Ania Filus, Jennifer Fraser, David Rowell, Aimee Johnston, Stephen Birch

**Affiliations:** School of Nursing, Midwifery and Social Work, The University of Queensland, St Lucia, Australia; Parenting and Family Support Centre, The University of Queensland, St Lucia, Australia; Centre for Mental Health, Griffith University, Mt Gravatt, Australia; Parenting and Family Support Centre, The University of Queensland, St Lucia, Australia; Australian Research Council Centre of Excellence for Children and Families over the Life Course, Brisbane, Australia; Dermatology Service, Queensland Children’s Hospital, Brisbane, Australia; Murdoch Children’s Research Institute, Parkville, Australia; Monash University, Melbourne, Australia; Parenting and Family Support Centre, The University of Queensland, St Lucia, Australia; Sydney Nursing School, University of Sydney, Sydney, Australia; Centre for the Business and Economics of Health, The University of Queensland, Brisbane, Australia; Centre for the Business and Economics of Health, The University of Queensland, Brisbane, Australia; Centre for the Business and Economics of Health, The University of Queensland, Brisbane, Australia

**Keywords:** adherence/self-management, health behavior, chronic illness, parenting, parents, randomized controlled trial

## Abstract

**Objective:**

To evaluate the efficacy and costs of a brief, group-delivered parenting intervention for families of children with eczema.

**Methods:**

A randomized controlled trial design was used. Families attending the Queensland Children’s Hospital and from the community (*n* = 257) were assessed for eligibility (child 2–10 years, diagnosed with eczema, prescribed topical corticosteroids). Families who consented to participate (*N *=* *59) were assessed at baseline for clinician-rated eczema severity, parent-reported eczema symptom severity, and electronically-monitored topical corticosteroid adherence (primary outcomes); and parenting behavior, parents’ self-efficacy and task performance when managing eczema, eczema-related child behavior problems, and child and parent quality of life (secondary outcomes). Families were randomized (1:1, unblinded) to intervention (*n *=* *31) or care-as-usual (*n *=* *28). The intervention comprised two, 2-hr *Healthy Living Triple P* group sessions (face-to-face/online) and 28 intervention families attended one/both sessions. All families were offered standardized eczema education. Families were reassessed at 4-weeks post-intervention and 6-month follow-up, with clinician-raters blinded to condition. Costs of intervention delivery were estimated.

**Results:**

Multilevel modeling across assessment timepoints showed significant intervention effects for ineffective parenting (*d* = .60), self-efficacy (*d* = .74), task performance (*d* = .81), and confidence with managing eczema-related child behavior (*d* = .63), but not disease/symptom severity, treatment adherence or quality of life. Mean cost per participating family with parenting behavior (clinically) improved was $159.

**Conclusions:**

*Healthy Living Triple P* is effective in reducing ineffective parenting practices and improving parents’ self-efficacy and task performance when managing children’s eczema and eczema-related behavior difficulties. There was no effect on disease/symptom severity, treatment adherence, or quality of life.

**Clinical Trial Registration:**

ACTRN12618001332213

Eczema is the most common chronic inflammatory skin condition worldwide (Harris & Cooper, 2017), and incidence among Australian children is among the highest in the world (Asher et al., 2006). Current pediatric guidelines (National Institute for Health Care Excellence, 2023) recommend daily application of emollient (moisturizer), even when eczema is clear; addition of topical corticosteroids for mild eczema; and addition of topical calcineurin inhibitors, bandaging (including “wet-wraps”), phototherapy and/or systemic therapy for moderate/severe disease. Parents’ ability to consistently implement their child’s treatment plan is key to successful management (Thompson & Thompson, 2014).

Despite this, two-thirds of children do not adhere to prescribed treatment (Krejci-Manwaring et al., 2007), increasing morbidity, healthcare utilization, and avoidable treatment escalation/failure (Bass et al., 2015). Treatment is time-consuming and demanding, and discomfort during treatment (e.g., stinging, burning, itching) can make treatment distressing (Santer et al., 2013; Teasdale et al., 2021). Children’s resistance to treatment (e.g., refusal to cooperate, complaining/arguing, tantruming) presents a significant problem, and can cause parents to shorten/omit aspects of treatment or skip treatment altogether (Burgess et al., 2008; LeBovidge et al., 2007; Penza-Clyve et al., 2004; Santer et al., 2013; Teasdale et al., 2021).

Many parents lack the self-efficacy (confidence) and effective parenting skills to manage difficult child behavior (Faught et al., 2007; Mitchell et al., 2015), and behavior problems and ineffective parenting practices are linked to poorer treatment adherence and worse health outcomes (Mitchell et al., 2015, 2016; Santer et al., 2014; Sokolova & Smith, 2015). This represents a vicious cycle, whereby eczema impacts child, parent, and family quality of life (Ablett & Thompson, 2016; Yang et al., 2019) and children’s development (Cheng & Silverberg, 2021; Pálsson et al., 2021; Yang et al., 2019), increasing risk of behavioral/adjustment problems (Yaghmaie et al., 2013) which are associated with poorer management and more severe eczema (Mitchell et al., 2015).

Parenting plays a central role in children’s health (Morawska et al., 2015; Wood et al., 2008). To date, most interventions for children with eczema have predominantly focused on education, and although some include psychosocial components (e.g., child/parent stress reduction) few have targeted parenting efficacy (Mitchell et al., 2020). Behavioral parenting interventions, recognized as best-practice (O'Connell et al., 2009), can improve parent self-efficacy, parenting behavior, illness severity/control, child quality of life and child behavior for families of children with chronic health conditions (Mitchell et al., 2020), and may be particularly relevant to eczema management where more effective parenting behavior and fewer child behavior problems are linked to better outcomes.

Trials of a group-delivered parenting intervention designed for families of children with a chronic health condition (*Healthy Living Triple P*) have demonstrated positive outcomes for families of children with asthma and/or eczema (Morawska et al., 2016, 2017a, 2017b), phenylketonuria (Mitchell et al., 2021a) and diabetes (Mitchell et al., 2022), including improved parent-reported eczema symptom severity, parenting behavior, parent self-efficacy, and child behavior. The main goal of eczema treatment is to reduce symptom and disease severity. Reducing obstacles to treatment adherence (e.g., child behavior and parenting difficulties) could therefore be expected to improve treatment adherence and thereby reduce symptom severity and improve disease control. However, effects on objectively-assessed disease severity and treatment adherence are yet untested.

This study evaluated the efficacy of a brief, group-delivered parenting intervention (*Healthy Living Triple P*) in improving child, parent, and family outcomes. We hypothesized that participation in *Healthy Living Triple P* would reduce eczema severity and improve treatment adherence (primary outcomes); and decrease ineffective parenting practices, increase parent self-efficacy with illness management, reduce child behavioral problems, and improve child and parent quality of life (QoL; secondary outcomes).

## Methods

A 2 (Triple P+education vs. care-as-usual [CAU] + education)×3 (time: baseline [T1], 4-week post-intervention [T2], 6-month follow-up [T3]) parallel design randomized controlled trial tested the relative impact of Triple P against CAU. Parents of 2- to 10-year-old children with eczema were recruited from dermatology outpatient clinics at the Queensland Children’s Hospital (QCH), and via advertisements and mail-outs to childcare centers, kindergartens, and primary schools in Brisbane, Australia. Inclusion criteria: (i) child, 2–10 years, with eczema; (ii) currently prescribed topical corticosteroids. Exclusion criteria: (i) child has a disability, including language/speech impairment; (ii) parents currently seeing a professional for child behavior difficulties; (iii) parents currently receiving psychological help/counseling or prior Triple P participation; or (iv) parents have difficulties in reading an English newspaper. The 2- to 10-year-old age group was selected as the *Healthy Living Triple P* intervention strategies are appropriate for use with children across this age group. Exclusion criteria were applied to ensure that the program would be appropriate for all children and parents and to reduce the risk of confounding due to concurrent professional intervention/counseling.

Ethical approval was granted by the Children’s Health Queensland (HREC/18/QRCH/28) and the University of Queensland (2018000449) Human Research Ethics Committees. The trial was prospectively registered (Australian New Zealand Clinical Trials Registration: ACTRN12618001332213) and followed the CONSORT guidelines. The CONSORT Checklist is available as Supplementary File 1.

From November 2018, families attending QCH received a study brochure along with their clinic appointment letter. Research assistant KK attended clinics to pre-screen for eligibility and provide written information/consent forms. Research coordinator A.E.M. phoned and screened interested families. Hospital-based recruitment was extremely slow; thus, recruitment was broadened (from November 2019) to include community-based families who responded to advertisements in school newsletters. Written parent consent and, where able, child assent were obtained by the research coordinator.

Arrival of COVID-19 in Australia resulted in hospital and community lockdowns from April 2020, which ended hospital-based recruitment and made community recruitment extremely difficult due to families’ lack of capacity during school closures. A decision was made to close recruitment in May 2020. Follow-up was completed in January 2021. No incentives were offered.

### Measures

Parents provided demographic/health history information (age at diagnosis, hospital separations, healthcare usage, comorbidities) at T1, and health information (diagnosis confirmation, medications, illness management plan/s) was requested from the dermatology team and/or GP (with parent consent). All other variables were assessed at T1, T2, and T3.

#### Measures—primary outcomes

**Disease and symptom severity.** The *Eczema Area Severity Index* (EASI; Tofte et al., 1998) is a visual clinical assessment measure of eczema disease severity. Higher scores indicate more severe disease (0–1.0 = clear/almost clear; 1.1–7.0 = mild; 7.1–21.0 = moderate; 21.1–50.0 = severe; 50.1–72.0 = very severe). EASI has good intra-rater reliability and sensitivity to change and is recommended by the Harmonising Outcome Measures for Eczema taskforce (Schmitt et al., 2014). Six experienced registered/clinical nurses each underwent 10+ hr of training and scoring practice with EC and/or AEM until adequate inter-rater reliability (intraclass correlation coefficient [ICC]≥.70) was achieved. A randomly-selected 20% of EASI assessments were done by 2 nurses to ensure ongoing reliability, which was excellent for in-person (ICC = .99) and photo-based (ICC = .92) assessments.

The *Patient-Orientated Eczema Measure* (POEM; Charman et al., 2004) measures patient- or caregiver-reported eczema symptoms. Parents report number of days during the previous week that skin has been (i) itchy, (ii) bleeding, (iii) weeping/oozing, (iv) cracked, (v) flaking, (vi) dry/rough; and (vii) disturbed night-time sleep (0 = no days, 1 = 1–2 days, 2 = 3–4 days, 3 = 5–6 days, 4 = every day). Item scores are summed, and higher total scores indicate more severe symptoms (0–2 = clear/almost clear; 3–7 = mild, 8–16 = moderate; 17–24 = severe; 25–28 = very severe). Internal consistency was good (*α* = .89).

**Treatment use and adherence.** Treatment use and adherence were monitored from 1 month prior to T1 assessments/randomization and continued until 6-month follow-up (7 months of monitoring). To assess use, topical corticosteroids and emollients were weighed (T1/T2/T3) and total use (g/day) calculated for each assessment interval. The *Medication Event Monitoring System* (MEMS) 6 TrackCap (AARDEX Group Ltd, Sion, Switzerland) was used to monitor topical corticosteroid adherence by recording dates/times corticosteroid tubes were accessed. Validity of the data collected from the MEMS TrackCaps and weighing of corticosteroids/emollients was assessed by comparing changes in weight to the TrackCap logs. In cases where change in weight was implausible (e.g., negative, no, or minimal weight change despite multiple openings recorded in the log) further information was sought to determine data accuracy (e.g., corticosteroids/emollients were reweighed, parents were questioned to ascertain the validity of data). In the few instances where inaccuracy was suspected, data were coded as missing.

Adherence was defined as at least daily use during the 7 days following EASI assessment if clinically indicated (EASI score mild/moderate/severe/very severe), and expressed as a percentage (e.g., use on 3/7 days = 42.9% adherence).

#### Measures—secondary outcomes

Parent-report online questionnaires were used to assess psychosocial outcomes (median completion = 29 min).

**Parenting behavior.** The 30-item *Parenting Scale* (Arnold et al., 1993) measures three dysfunctional discipline styles: laxness, over-reactivity, and verbosity. Parents rate their usual behavior using a 7-point scale, and mean item scores provide subscale and total scores, compared against published clinical cut-offs. Internal consistency was adequate for Laxness (*α* = .83), Overreactivity (*α* = .86) and the total score (*α* = .84), but low for Verbosity (α = .19) which was excluded from analyses.

**Eczema management.** The 25-item *Child Eczema Management Questionnaire* (Mitchell & Fraser, 2011) assesses parents’ self-efficacy and self-reported task performance with eczema management. For each eczema management task, parents rate their (a) confidence (Self-Efficacy) with performing that task, 0 (*certain I can’t do it*) to 10 (*certain I can do it*), and (b) how often they do that task successfully (Task Performance), 0 (*never*) to 10 (*always*). Scale scores (mean item scores) range from 0 to 10, with higher scores indicating greater self-efficacy and better task performance. Reliability was excellent (Self-Efficacy *α* = .95, Task Performance *α* = .97).

**Child behavior.** The 25-item *Eczema Behaviour Checklist* (Mitchell et al., 2017) assesses extent of child behavior difficulties related to eczema management, 1 (*not at all*) to 7 (*very much*), and of parents’ confidence with managing these, 1 (*certain I can’t do it*) to 10 (*certain I can do it*). Higher Extent (25–175) scores and Confidence (25–250) scores indicate greater child behavior difficulties and greater self-efficacy with managing difficult child behaviors, respectively. Extent (*α* = .94) and Confidence (*α* = .98) had excellent reliability.

**Quality of life.** Impact of eczema on children’s QoL was assessed using the 10-item child self-report *Child Dermatology Life Quality Index* (Lewis-Jones & Finlay, 1995) for children aged 4–10 years (*α* = .84), and the 10-item parent-report *Infants’ Dermatitis Quality of Life Index* (Lewis-Jones et al., 2001) for children aged 2–3 years (*α* = .82). All parents completed the 10-item *Dermatitis Family Impact Questionnaire* (Lawson et al., 1998) to assess impact of eczema on family QoL (*α* = .92). Scores for all three scales range from 0 to 30, allowing child and infant scores to be combined into a single quality of life variable, and higher scores indicate greater impact on QoL.

**Intervention acceptability.** The *Client Satisfaction Questionnaire* (Sanders et al., 2001) is a 13-item measure of parents’ program satisfaction. Ten items assess perceptions of program quality, extent to which families’ needs were met, and effectiveness in improving parenting skills and child behavior using a 1–7 scale. Total scores range from 10 to 70, with higher scores indicating greater satisfaction.

**Economic evaluation.** An economic evaluation of *Healthy Living Triple P* was undertaken using a cost consequence analysis (Drummond et al., 2015). This method can be used to measure intermediate impacts that might be expected to lead to health outcomes and provides a measure of the return of investment in the program. Costs of achieving clinically significant improvement ineffective parenting practices (the consequence of interest, or quasi outcome) were identified.

### Procedure

Families were assessed at baseline (T1), then randomized to intervention or CAU (1:1) using computer-generated randomly-selected block sizes (4, 6 or 8 families per block) and random group allocation within blocks. An external researcher generated the random allocation sequence, using sequentially numbered, opaque, sealed envelopes to conceal group allocation from researchers and participants until after completion of all baseline assessment. The research coordinator (AEM) allocated envelopes to families in order of baseline assessment completion and informed families of their allocation. Parents allocated to intervention were booked into Triple P sessions (2×2-hr group sessions). Families allocated to CAU were invited to attend the sessions after completing the study.

Families received 4 home visits. At enrolment (Visit 1), a research assistant (EF) weighed and recorded children’s usual topical treatments in a paper diary (which parents kept to record treatment use throughout the study) and set-up MEMS TrackCap monitoring. After 4 weeks, registered nurses (not involved in children’s care, blinded to group allocation) re-weighed and recorded topical treatments, conducted baseline (T1) EASI assessments, and administered the infant/child QoL questionnaires (Visit 2). This was repeated at 4-weeks post-intervention (Visit 3) and at 6-month follow-up (Visit 4). The same nurse conducted all 3 EASI assessments wherever possible.

Disruptions due to COVID-19 necessitated changes to assessment procedures from March 2020 onwards. Home visits were replaced by Zoom videoconferences. Digital scales and monitoring equipment were mailed to families, and EF coached parents through the set-up of diaries and MEMS TrackCaps in real-time. In-person EASI assessments were replaced by photo-based EASI assessments, with parents uploading high-resolution digital images via a secure online file transfer system. Images were scored by the EASI trainers (AEM and EC).

#### Parenting intervention

*Healthy Living Triple P* is designed for parents of children with chronic health conditions. It comprises two, 2-hr group discussion sessions that draw on the theoretical principles underpinning the Triple P-Positive Parenting Program, including social learning, cognitive behavioral and developmental theory (Sanders, 2023). It aims to increase parenting skills and confidence, parental self-regulation and use of effective parenting practices to support strong parent–child relationships and children’s emotional/behavioral adjustment. The program is interactive, with opportunities for discussion, and flexible enough to meet the differing needs of parents of 2- to 10-year-old children.

Session 1 focuses on preventing and managing problems associated with children’s health conditions. It introduces strategies that empower parents to prevent and manage problems with condition management, reduce the impact of the child’s condition, and ensure that prevention and management plans are implemented appropriately. These include having realistic expectations, reducing families’ and children’s stress and anxiety, involving children in their own eczema treatment, and monitoring symptoms.

Session 2 introduces principles of positive parenting to support caring parent–child relationships, promote positive parenting practices, and the use of assertive and consistent discipline strategies. It empowers parents to identify contributors to child behavior difficulties (e.g., child/family stress, inconsistent parenting, symptom-related factors) and develop plans for managing difficult behavior effectively. Session delivery is tailored to families’ specific needs, and families draw on their own experiences to problem-solve and develop plans for good eczema management that support child, parent, and family health, relationships and wellbeing.

#### Education intervention

All families were provided with standardized eczema education delivered by dermatology nurse practitioner EC as part of usual care at QCH and/or via a 30-min online eczema education module (developed by EC) comprising 4 short videos that deliver identical content to the hospital education session. The education session/module provides up-to-date, evidence-based information about eczema, including etiology, signs and symptoms, triggers, treatment options, and management tips and strategies. Links to the online eczema education module were emailed to all intervention group parents following Session 1, and families allocated to CAU received access 1 week after randomization. All families had access to standardized eczema education at least 5 weeks prior to T2 assessment.

### Protocol adherence

Four accredited Triple P practitioners (2 psychologists, 1 social worker, 1 pediatric nurse) delivered *Healthy Living Triple P* according to a standardized manual. Practitioners completed protocol adherence checklists, and video-recordings of 25% of group sessions (≥2 sessions/practitioner) were coded for protocol adherence: 100% of content was covered, with 100% agreement between facilitators and an independent rater.

### Statistical analyses

We estimated that 150 participants were needed (*α* = .05 and *β* = .80) to detect a medium effect size (Cohen’s *d* = .50) for symptom severity while allowing for 20% attrition, consistent with earlier data (Morawska et al., 2016, 2017a, 2017b). In all, 10.2% of data were missing completely at random; the full information maximum likelihood approach allowed inclusion of all cases. Intention-to-treat (ITT) analyses were conducted using SPSS v27. Mixed-model repeated measures (MMRM) hierarchal linear models (multilevel modeling) compared change over time for intervention compared to CAU on primary and secondary outcomes across all 3 assessment timepoints (baseline, 4-week post-intervention, 6-month follow-up). After assessing for a main effect of time (fixed and random effects; Model 1), fixed effects of group and group-by-time interactions were added (Model 2) with statistically significant group-by-time interaction terms indicating intervention effects. Follow-up contrasts using fixed and random effects of time as predictors were run separately for intervention (Model 3) and CAU (Model 4) groups to interpret the direction of intervention effects, and *t*-tests compared rate of change (regression parameter estimate for time) between groups. Cohen’s *d* indicated effect size (Cohen, 1988). Proportions of families showing clinically important and statistically reliable change were assessed via (i) published minimum clinically important difference scores for EASI and POEM, (ii) movement from clinical to nonclinical ranges on the Parenting Scale, and (iii) reliable change indices for the Parenting Scale (Jacobson & Truax, 1991).

### Economic evaluation

Costs were based on actual charges or quotes from vendors and assessed from the perspective of QCH using a top-down approach, with *Healthy Living Triple P* delivered to 31 participants across 4 groups (i.e., 8 participants/group, maximum capacity 12 participants/group), and an additional 2 hrs’ labor to open/close each session. The cost of practitioner time ($59.77/hr at Queensland Health pay rates) covered both direct payment to the practitioner and additional employment costs incurred by the employer (e.g., superannuation). Costs for use of a QCH seminar room ($300/day, quoted by QCH), participant workbooks ($5.87/book, quoted by University printery), and practitioner training and accreditation ($1,255/practitioner, charged by Triple P International) were included. Because the evaluation was limited to a 6-month follow-up period, there was no need to discount costs and effects to allow for any differences in the distribution of effects and costs of longer periods.

The estimated costs of a scaled-up program based on a steady state of program capacity and current staff complements were calculated. During 2019, 501 eligible families were referred to the QCH dermatology outpatient clinic. All parents are invited to attend the program; however, experience shows that uptake is usually well below 100%. Modeling of future costs was therefore based on 3 recruitment scenarios, with full capacity expected to include 300 participants, and 75% and 50% capacity utilization including 225 and 150 participants, respectively.

### Data availability

Given that the data collected in this project are considered sensitive, deidentified data will only be made conditionally available with approval from the original research team.

## Results

### Sample

**Figure 1.**
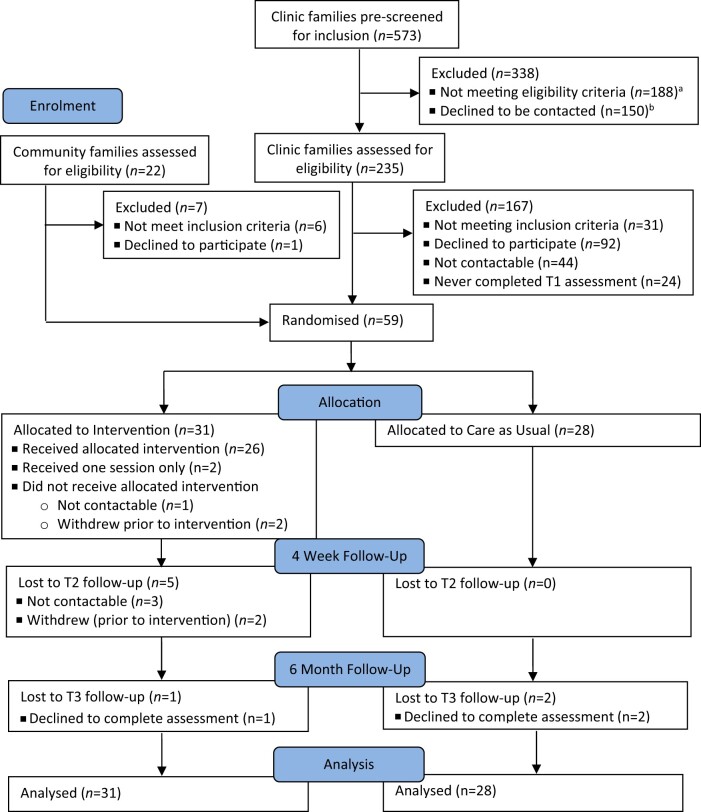
CONSORT diagram of flow of participants. ^a^Clinically ineligible families screened out by clinic staff (no eczema diagnosis, *n* = 100; not prescribed topical corticosteroids, *n* = 8); another 80 families excluded by the research assistant (insufficient English, *n* = 30; lived too far away, *n* = 27; child intellectual/developmental disability, *n* = 14; parents seeing psychologist, *n* = 4; previously completed Triple P, *n* = 2; child in foster care, *n* = 3). ^b^Declined to be contacted (uninterested, *n* = 44; no time, *n* = 38; no concerns, *n* = 19; eczema “too mild”, *n* = 16; travel difficulties, *n* = 18; caregiving responsibilities, *n* = 9; unable to attend sessions, *n* = 3; no reason, *n* = 3).

Overall, 573 families of children aged 2–10 years attended QCH eczema outpatient clinics between November 2018 and March 2020; 388 families were screened out as ineligible or unavailable (see Figure 1). Of the remaining 235 families who expressed interest and consented to be contacted, 191 were contactable (3 attempts to contact via phone plus voicemail/email). Of these, 31 did not meet inclusion criteria (previously completed Triple P, *n *=* *9; currently seeing psychologist, *n *=* *6; child intellectual/developmental disability, *n *=* *6; not prescribed topical corticosteroids, *n *=* *3; lived too far away, *n *=* *3; insufficient English, *n *=* *2; no more eczema, *n *=* *1; child turned 11, *n *=* *1), resulting in 160 eligible families. Of these, 92 declined (no time, *n *=* *42; no concerns, *n *=* *24; not interested, *n *=* *12; caregiving demands, *n *=* *5; travel difficulties, *n *=* *3; COVID-19-related stress, *n *=* *2; unable to attend sessions, *n *=* *1; no reason, *n *=* *3), and 24 never consented. Forty-four families (27.5%) consented to participate, completed baseline assessment and were randomized into the study.

An additional 22 families from the community contacted the research team and were screened. Six were ineligible (not prescribed topical corticosteroids, *n *=* *2; child autism spectrum disorder, *n *=* *2; already done Triple P, *n *=* *1; child too young, *n *=* *1), but 16 were eligible and 15 (93.8%) consented, completed baseline assessment, and were randomized into the study. Only 1 eligible parent declined to participate (citing COVID-19-related stress). The final sample (*n *=* *59) completed T1 assessment and were randomly allocated to intervention (*n *=* *31) or CAU (*n *=* *28). Recruitment stopped early due to slow recruitment and conclusion of study funding. Participant progress through study is detailed in Figure 1.

Parent participants were predominantly mothers, university educated, working full- or part-time and in a couple relationship (see Table 1). The proportion with a university degree (71.2%) was higher than for Australian women aged 35–44 (49.7%; Australian Bureau of Statistics, 2023a) but the proportion in paid employment (78.0%) was similar to that of Australian mothers in 2-parent families with dependent children (76.4%; Australian Bureau of Statistics, 2023c). The proportion of children living in their original family (84.7%) was higher than for children aged 0–14 years in Australia (72%; Australian Bureau of Statistics, 2023b).

**Table 1.** Demographic and clinical characteristics of participants by group.

Variables		Intervention (*n *=* *31)	Care as Usual (*n *=* *28)	
		*M* (*SD*)	*M* (*SD*)	*t*
Parent’s age (years)		38.35 (5.23)	36.89 (5.61)	−1.04
Child’s age (years)		5.42 (2.88)	5.00 (2.06)	−0.65
Child’s age at diagnosis (years)		1.24 (1.67)	1.04 (1.00)	−0.54
Clinician-rated eczema severity	EASI	5.47 (6.27)	5.21 (6.24)	−0.15
		
		% (*n*)	% (*n*)	*χ* ^2^
		
	Clear/almost clear	12.9 (4)	14.3 (4)	–
	Mild	67.7 (21)	67.9 (19)	
	Moderate	9.7 (3)	7.1 (2)	
	Severe	6.5 (2)	7.1 (2)	
	Very severe	*-*	–	
	Not assessed	3.6 (1)	3.6 (1)	
		*M* (*SD*)	*M* (*SD*)	*t*
Parent-report symptom severity	POEM	12.87 (8.96)	11.04 (6.36)	−0.91
		
		% (*n)*	% (*n*)	*χ^2^*
		
	Clear/almost clear	9.7 (3)	7.1 (2)	–
	Mild	29.0 (9)	21.4 (6)	
	Moderate	25.8 (8)	50.0 (14)	
	Severe	22.6 (7)	17.9 (5)	
	Very severe	12.9 (4)	3.6 (1)	
Child’s sex	Male	41.9 (13)	50.0 (14)	0.13
	Female	58.1 (18)	50.0 (14)	
Household	Original family	83.9 (26)	85.7 (24)	–
	Sole parent/step-family	12.9 (4)	10.7 (3)	
	Other	3.2 (1)	3.6 (1)	
Parent’s relationship to child	Mother	96.8 (30)	82.1 (23)	–
	Father	3.2 (1)	17.9 (5)	
Parent’s education	High school or less	9.7 (3)	14.3 (4)	–
	Trade/college	12.9 (4)	21.4 (6)	
	University degree	25.8 (8)	35.7 (10)	
	Postgraduate degree	51.6 (16)	28.6 (8)	
Relationship status	Married/de facto	83.9 (26)	89.3 (25)	–
	Separated/divorced/single	16.2 (5)	10.7 (3)	
Ethic/cultural identity^b^	Asian	35.5 (11)	39.3 (11)	–
	Oceanian	16.1 (5)	28.6 (8)	
	European	16.1 (5)	17.9 (5)	
	African	3.2 (1)	3.6 (1)	
	South American	–	3.6 (1)	
	Not specified	29.0 (9)	7.1 (2)	
Parent’s employment	Full-time/part-time	83.9 (26)	71.4 (20)	–
	Job-seeking	9.7 (3)	3.6 (1)	
	Not working	6.5 (2)	25.0 (7)	
Meeting essential expenses^a^	Yes	77.5 (24)	92.9 (26)	–
	No	22.6 (7)	7.1 (2)	
After expenses can afford	Not much	32.3 (10)	17.9 (5)	1.99
	Some things	29.0 (9)	42.9 (12)	
	Most things	38.7 (12)	39.3 (11)	

*Note.* EASI = Eczema Area Severity Index (0–1.0 = clear/almost clear; 1.1–7.0 = mild; 7.1–21.0 = moderate; 21.1–50.0 = severe; 50.1–72.0 = very severe); POEM = Patient Oriented Eczema Measure (0–2 = clear/almost clear; 3–7 = mild, 8–16 = moderate; 17–24 = severe; 25–28 = very severe).

a Able to meet living expenses over past 12 months.

b Based on Australian Bureau of Statistics Standard Classification of Cultural and Ethnic Groups, 2019.

There were no substantial differences between groups at baseline (Table 1). Children were diagnosed from birth to 7 years, although most (72.9%) were diagnosed by age 1. All children were prescribed topical corticosteroids and emollients by their treating clinicians. Baseline EASI scores (nurse-rated) classified most children’s eczema as clear/almost clear (14.0%, *n = *8) or mild (70.2%, *n *=* *40); few had moderate (8.8%, *n *=* *5) or severe (7.0%, *n *=* *4) disease (EASI *M *=* *5.35, *SD *=* *6.20, range 0.2–27.2). POEM scores (parent-rated) classified children as having eczema that was clear/almost clear (8.5%, *n *=* *5), mild (25.4%, *n *=* *15), moderate (37.3%, *n *=* *22), severe (20.3%, *n *=* *12) or very severe (8.5%, *n *=* *5) (POEM *M *=* *12.00, *SD *=* *7.82).

### Intervention use and attrition

Triple P sessions were held face-to-face at QCH from March 2019 to March 2020, then via Zoom from April to August 2020, due to COVID-19. Discussion groups (6 face-to-face, 7 online) comprised an average 4 parents from 3 families. Twenty-six families (83.9%) attended both Triple P sessions, 2 (6.5%) attended one session, and 3 (9.7%) attended none (not contactable, *n *=* *1; withdrew prior to intervention, *n *=* *2). Attendance at Zoom sessions (100%) was better than at face-to-face sessions (87.2%). Ten families (38.5%) had a second parent (all fathers) attend in addition to the primary caregiver. Usage of the online eczema education module was tracked via Qualtrics: 7 families (11.9%) attended the hospital-based education session prior to randomization and did not access the online module, 5 families (8.5%) accessed the online module only, and 47 families (79.7%) accessed neither form of education.

All families who attended both Triple P sessions completed T2 assessment; the 2 families who had only attended 1 session did not complete T2 assessment; and 1 further family declined T3 assessment. Of families allocated to CAU, all were retained to T2, but 2 families declined T3 assessment. All families who declined assessments cited lack of time and/or stress related to COVID-19 as reasons. No adverse effects, concerns, or unintended consequences were identified.

### Intervention effects

Means, standard deviations, and effect sizes (Cohen’s *d*) are presented in Table 2. Results of MMRM analyses assessing rate of change across all assessment timepoints (T1–T3) are presented in Table 3. Visual depictions of rate of change over time based on regression parameters are presented in Supplementary Figures S1 and S2. Clinically significant improvements in disease (EASI) and symptom (POEM) severity, and clinically significant and reliable change on the Parenting Scale, are shown in Supplementary Tables S1 and S2, respectively.

**Table 2.** Means and standard deviations for primary and secondary outcomes by treatment condition, and effect sizes.

	Intervention^a^			Care as Usual^b^			Effect size^c^	
	T1	T2	T3	T1	T2	T3	T1–T2	T1–T3
Measure	*M*	*M*	*M*	*M*	*M*	*M*	*d*	*d*
	(*SD*)	(*SD*)	(*SD*)	(*SD*)	(*SD*)	(*SD*)	[95% CI]	[95% CI]
Primary outcomes								
Eczema severity								
Clinician-rated	5.47	6.77	4.73	5.21	6.32	5.13	0.07	0.22
	(3.70)	(3.10)	(3.70)	(3.80)	(3.10)	(3.10)	[−0.49, 0.64]	[−0.36, 0.80]
Parent-report	12.87	12.48	9.52	11.04	9.92	9.35	−0.05	0.26
	(8.96)	(7.29)	(7.74)	(6.36)	(6.63)	(6.32)	[−0.60, 0.51]	[−0.31, 0.82]
Treatment use								
Emollient use (g/day)	16.53	14.05	7.85	10.76	8.51	7.88	−0.02	−0.32
	(21.18)	(15.70)	(11.67)	(13.49)	(9.47)	(9.72)	[−0.61, 0.56]	[−0.94, 0.30]
Steroid use (g/day)	0.84	0.88	0.87	0.69	0.46	0.54	0.38	0.18
	(0.90)	(0.98)	(1.17)	(0.49)	(0.33)	(0.82)	[−0.21, 0.98]	[−0.44, 0.81]
Adherence (% days)^d^	45.00	30.92	–	43.38	50.40	–	−0.35	–
	(37.20)	(35.26)		(39.81)	(34.83)		[−1.09, 0.39]	
Secondary outcomes								
Parenting practices								
Ineffective parenting	3.55	2.87	2.91	3.05	3.04	3.05	**0.84**	**0.60**
	(0.79)	(0.70)	(0.71)	(0.63)	(0.57)	(0.59)	**[0.26, 1.42]**	**[0.03. 1.18]**
Laxness	3.23	2.33	2.14	2.74	2.73	2.73	**0.66**	**0.61**
	(1.25)	(0.69)	(0.68)	(0.98)	(0.94)	(0.91)	**[0.09, 1.23]**	**[0.03, 1.19]**
Overreactivity	3.26	2.73	2.96	2.51	2.59	2.70	0.44	0.44
	(1.18)	(1.11)	(1.15)	(0.97)	(0.83)	(1.02)	[−0.13, 1.00]	[−0.13, 1.02]
Eczema management								
Self-efficacy	7.40	7.82	8.36	8.37	8.30	8.35	0.47	**0.74**
	(1.51)	(1.62)	(1.64)	(1.19)	(1.48)	1.41	[−0.09, 1.04]	**[0.16, 1.32]**
Task performance	6.83	7.38	7.99	8.13	8.14	8.23	0.56	**0.81**
	(1.66)	(1.85)	(2.26)	(1.25)	(1.55)	1.37	[−0.01, 1.13]	**[0.22, 1.40]**
Eczema behavior								
Extent	83.32	72.05	65.71	72.75	70.08	66.68	0.27	0.52
	(34.82)	(32.13)	(38.69)	(33.25)	(40.18)	(32.45)	[−0.30, 0.83]	[−0.06, 1.11]
Confidence	178.25	200.00	217.34	211.25	208.64	211.42	0.26	**0.63**
	(54.21)	(34.14)	(38.76)	(41.67)	(43.72)	(34.93)	[−0.33, 0.84]	**[0.01, 1.25]**
Quality of life								
Child	8.10	7.73	6.32	7.64	7.62	6.44	0.15	0.25
	(5.71)	(6.16)	(5.86)	(5.03)	(5.95)	(4.31)	[−0.39, 0.68]	[−0.28, 0.79]
Family	11.16	10.23	8.95	10.46	9.44	6.94	0.03	0.08
	(7.68)	(8.33)	(7.84)	(7.07)	(7.95)	5.60	[−0.53, 0.60]	[−0.49, 0.65]

*Note.* Bolded figures indicate statistically significant effect sizes.

a Intervention group T1 *n *=* *31, T2 *n *=* *23, T3 *n *=* *21.

b CAU group T1 *n *=* *28, T2 *n *=* *25, T3 *n *=* *26.

c Effect size represents the pre-post change in intervention group minus the pre-post change in Care as Usual group, divided by the pooled baseline *SD* and corrected for bias.

d For children with mild/moderate/severe/very severe eczema (EASI score >1.0) at T1 and T2 only; intervention group T1 *n *=* *13, T2 *n *=* *12; CAU group T1 *n *=* *16, T2 *n *=* *15.

**Table 3.** Intervention effects for primary and secondary outcome variables.

Model	1				2				3		4			
	Estimate of fixed effects: time^a^				Estimate of fixed effects: time × condition interaction^a^^,b^				Estimate of fixed effects: time (separate by condition)^a^				Comparison^c^	
									Intervention		Care as Usual			
Measure	*B*	*F*	*df*	*p*	*B*	*F*	*df*	*p*	*B*	*p*	*B*	*p*	*t*	*p*
Primary outcomes														
Eczema severity														
Disease severity	−0.18	0.15	58.17	.696	−0.47	0.25	58.09	.619	−0.52	.331	0.07	.934	0.62	.538
Symptom severity	−1.08	5.90	52.48	**.019**	−0.76	0.74	52.05	.394	−1.46	.060	−0.68	.194	0.86	.392
Treatment use														
Emollient use (g/day)	−2.92	6.90	53.06	**.001**	−2.91	1.77	52.95	.189	−4.37	**.035**	−1.46	.152	1.32	.192
Steroid use (g/day)	−0.03	0.17	49.78	.684	0.07	0.21	49.81	.650	<0.01	.971	−0.07	.443	0.80	.429
Adherence (% days)	5.38	0.40	26.93	.534	−16.85	1.74	27.01	.198	−10.93	.197	5.62	.560	1.34	.095
Secondary outcomes														
Parenting practices														
Ineffective parenting	−0.14	9.35	42.45	**.004**	−0.29	10.99	43.82	**.002**	−0.30	**.002**	<−0.01	.919	3.30	**.002**
Laxness	−0.24	12.11	41.02	**.001**	−0.47	14.60	43.82	**<.001**	−0.52	**<.001**	−0.01	.811	4.07	**<.001**
Overreactivity	−0.03	0.26	49.73	.609	−0.26	5.95	49.88	**.018**	−0.17	.096	0.10	.089	2.37	**.021**
Eczema management														
Self-efficacy	0.24	6.15	50.31	.**017**	0.50	7.60	49.60	**.008**	0.50	**.007**	<0.01	.972	−2.60	**.012**
Task performance	0.34	9.56	49.89	**.003**	0.62	9.93	49.31	**.003**	0.68	**.002**	0.05	.605	−2.93	**.005**
Eczema behavior														
Extent	−5.38	6.48	49.51	**.014**	−8.25	4.11	48.56	**.048**	−9.30	**.019**	−1.54	.484	1.81	.075
Confidence	7.99	7.14	42.99	**.011**	17.10	9.55	43.38	**.003**	15.38	**<.001**	−0.59	.809	−3.48	**<.001**
Impact on quality of life														
Child	−0.73	4.90	55.13	**.031**	−0.59	0.81	54.93	.371	−1.07	**.018**	−0.43	.398	0.97	.335
Family	−1.54	13.06	52.52	**.001**	0.25	0.08	52.54	.775	−1.34	.098	−1.63	**.001**	−0.32	.749

*Note.* Intention-to-treat analyses (Intervention *n *=* *31, Care as Usual *n *=* *28). Bold values indicate statistically significant outcomes.

a *B* = estimated regression coefficients from mixed-model repeated-measures linear regressions.

b Figures indicate the estimated change in the intervention group from pre-intervention to post-intervention follow-up relative to the Care as Usual group.

c Test of the difference between Intervention and Care as Usual groups for rate of change (estimated regression coefficients); degrees of freedom = 55 for all models except Adherence, where degrees of freedom = 33.

#### Intervention effects—primary outcomes

**Disease and symptom severity.** Time did not predict change in disease severity (EASI; Table 3), and lack of time-by-group interaction indicated no statistically significant group-level intervention effect (small effect *d = *0.22 at T3; Table 2). Proportions of children showing clinically important change in disease severity (change of ≥6.6 points on EASI) from T1–T2 and T1–T3 were similar between groups (Supplementary Table S1).

In contrast, time did predict change (improvement) in parent-reported eczema symptom severity (POEM; Table 3); however, there was no statistically significant intervention effect (small effect *d *=* *0.26 at T3; Table 2). Proportions of parents reporting clinically important improvement/worsening in symptoms (change of ≥3 points on POEM) from T1–T2 and T1–T3 were similar between groups (Supplementary Table S1).

**Treatment use and adherence.** Time predicted change (decrease) in emollient use but lack of time-by-group interactions indicated no statistically significant intervention effect. Time did not predict change in topical corticosteroid use, or change in topical corticosteroid adherence for the subsample of children (91.5%, 54/59) with mild/moderate/severe/very severe disease (Table 3), with small effect sizes (*d* = -0.32–0.38; Table 2).

#### Intervention effects—secondary outcomes

**Parenting behavior.** Time predicted change (decrease) in use of ineffective parenting practices (Parenting Scale Total), and a statistically significant time-by-group interaction indicated a medium-large intervention effect (*d* = .84 at T2, *d* = .60 at T3). Follow-up contrasts showed greater rate of change for intervention than CAU, which showed no change. When scores were examined by subscale, statistically significant time-by-group interactions for Laxness and Overreactivity indicated small-medium intervention effects (Table 2). Follow-up contrasts (Table 3) showed a greater rate of decrease for intervention than CAU, which showed no change.

Overall, 53.8% (7/13) of parents in the intervention group who were in the clinical range at T1 moved into the nonclinical range by T2, compared to 12.5% (1/8) of those in CAU (Supplementary Table S2). By T3, 54.5% (6/11) of parents in the intervention group who were in the clinical range at T1 had moved into the nonclinical range, compared to none (0.0%, 0/8) of those in CAU. Chi-squared tests for independence indicated a greater proportion of participants in the intervention group showed reliable improvement in Total and Laxness scores from T1–T3 than CAU. There was no association between group and reliable worsening on any measures.

**Eczema management.** Time predicted improved parent Self-Efficacy and Task Performance (Table 3), and significant time-by-group interactions indicated medium-large intervention effects (*d = *0.47 and 0.56 at T2; *d *=* *0.74 and 0.81 at T3 for Self-Efficacy and Task Performance, respectively). Follow-up contrasts showed greater rates of improvement for the intervention group compared to CAU, which showed no change.

**Eczema-related child behavior.** Time predicted change (improvement) in behavior difficulties (Extent scores) and parents’ confidence with managing them (Confidence scores). Time-by-group interactions indicated small-medium intervention effects (*d *=* *0.27 and 0.26 at T2; *d *=* *0.52 and 0.63 at T3 for Extent and Confidence, respectively). Follow-up contrasts showed within-group improvements for Extent and Confidence for the intervention group compared to no change for the CAU group on either measure, although the difference between the groups only attained statistical significance for Confidence.

**Quality of life.** Time predicted change (reduction) in impact of eczema on child and family QoL; however, lack of group-by-time interactions indicated no intervention effects (Table 2).

**Intervention acceptability.** Parents (*n *=* *21) who completed the Client Satisfaction Survey rated session quality as excellent (*M *=* *6.33, *SD *=* *0.80) and were satisfied/very satisfied with the program (*M *=* *6.00, *SD *=* *1.00). Most received the type (90.5%, *n *=* *19) and amount (90.5%, *n *=* *19) of help they wanted, and felt that the program helped them to deal more effectively with child behavior (90.5%, *n *=* *19), eczema management (81.0%, *n *=* *17), and family problems (81.0%, *n *=* *17). Satisfaction scores were similar for families who attended sessions in-person (*n *=* *11, *M *=* *55.55, *SD *=* *8.94) or online (*n *=* *10, *M *=* *54.40, *SD *=* *8.33).

**Economic evaluation.** The recurrent cost of providing the intervention to 4 groups of parents was AUD$4,060 (see Supplementary Table S3). An already-accredited Triple P practitioner could deliver *Healthy Living Triple P* to families, avoiding additional training costs. Ongoing intervention costs were modeled on the assumption that staff are already trained and accredited in Triple P. Ongoing costs are modeled based on 3 recruitment scenarios (Table 4). The cost is AUD$87 per participant under all recruitment scenarios, assuming that each group runs at capacity.

**Table 4.** Modeled costs for ongoing *Healthy Living Triple P* intervention delivery.

Cost categories	Description	Cost per unit (AUD 2019)	# Units	Total cost (AUD 2019)
Recurrent costs				
* *100% capacity utilization: 300 participants in 25 groups				
Facilitation	8 hr Practitioner wages (plus 40% on costs)	$670 per group	25 groups	$16,750
Capital	Seminar room @ QCH	$300 per day	50, half-days	$7,500
Consumables	Parent workbooks	$5.87 per workbook	300 books	$1,761
Total recurrent costs				**$26,011**
75% capacity utilization: 225 participants in 19 groups				
Facilitator wages	8 hr Practitioner wages (plus 40% on costs)	$670 per group	19 groups	$12,730
Capital	Seminar room @ QCH	$300 per day	38, half-days	$5,700
Consumables	Parent workbooks	$5.87 per workbook	225 books	$1,321
Total recurrent costs				**$19,751**
* *50% capacity utilization: 150 participants in 13 groups				
Facilitator wages	8 hr Practitioner wages (plus 40% on costs)	$670 per group	13 groups	$8,710
Capital	Seminar room @ QCH	$300 per day	26, half-days	$3,900
Consumables	Parent workbooks	$5.87 per workbook	150 books	$881
Total recurrent costs				**$13,491**

*Note.* QCH = Queensland Children’s Hospital. The nominated cost of a seminar room at QCH was quoted at $300/day by the Finance Department. Capital costs assume that groups are run at full capacity. Bold values are total costs.

Although no statistically significant effect was observed for the primary outcome measures, statistically significant change in parenting practices, an important secondary outcome measure, was observed. *Healthy Living Triple P* reduced ineffective parenting (parents with PS Total scores in the clinical range) by 54.5% (*p* =.018) by 6-month follow-up (T1–T3). Hence, at full capacity with a cohort of 300 participants, the intervention is estimated to reduce ineffective parenting in 164 participants. The mean cost per participating parent with parenting behavior (clinically) improved would be AUD$159 (AUD$26,011/164).

## Discussion

This study is one of the first to examine the effects of a brief parenting support program on clinician-assessed disease severity and objectively-measured treatment adherence alongside indicators of parent and child behavior and adjustment. Although results were mixed, they contribute to understanding the role of parenting support in pediatric settings and provide direction for future research and clinical practice.

As hypothesized, *Healthy Living Triple P* reduced ineffective parenting practices and improved task performance and self-efficacy when managing children’s eczema and eczema-related behaviors at 6-month follow-up, with medium-sized effects. These results align with previous studies testing parenting interventions in the pediatric chronic illness context (Mitchell et al., 2020) including earlier trials of *Healthy Living Triple P* (Morawska et al., 2016, 2017a, 2017b). Importantly, half of families in the clinical range for ineffective parenting at baseline moved into the nonclinical range by 6 weeks post-intervention and sustained improvement to 6-month follow-up compared to no parents in the CAU group, a result that mirrors findings from a previous trial (Morawska et al., 2017b) and adds data in support of intervention efficacy for these important parent outcomes.

There was no effect on clinician-rated disease severity. Despite recruiting via a major pediatric tertiary referral center, most children (84.2%) had mild eczema. This made it impossible for most to achieve clinically significant improvement on the EASI (change ≥6.6 points), and although trends were in the expected directions our study was underpowered to detect such small changes. Restricting inclusion to children with moderate/severe/very severe disease (EASI ≥7.1 points) may be needed to establish clinically significant effects.

Likewise, there was no effect on parent-reported symptom severity. This contrasts with a previous trial of this intervention with a community-recruited sample of parents of children with eczema and/or asthma, which found a large effect on parent-reported eczema symptom severity (*d *=* *0.95), although only for the subsample of children who were prescribed topical corticosteroids (Morawska et al., 2016). There are several potential explanations for the conflicting results from these 2 trials. First, parent-reported symptom severity was lower in this sample compared to the previous study, providing less scope for improvement. Second, the current trial administered POEM as a single-point-in-time retrospective rating of eczema symptoms over the previous week, whereas the previous trial had parents complete a daily symptom checklist from which data were extracted to calculate the POEM score. The use of a daily symptom checklist may have caused parents to monitor their child’s skin more closely—indeed, parents would have needed to undertake a daily visual assessment of their child’s skin to complete the checklist. This may have affected parents’ use of and/or adherence to treatment, and perhaps even triggered parents to initiate treatment (e.g., applying creams or wet-wraps) depending on the condition of their child’s skin.

Third, eczema (medical management) education was delivered in different ways across the trials. The previous trial delivered brief eczema education to the intervention group only, via a printed tip sheet and a video demonstrating application of topical treatments and wet-wraps. These resources were fully integrated into the Triple P sessions: the video was played during Session 1, followed by a brief group discussion about treatment use, and parents typically read the tip sheet during or between sessions. The current trial provided all parents (intervention and CAU) with comparable eczema education content, albeit in greater detail and in an online video format that families could access at any time. However, use of the online eczema education modules was extremely low, and although some parents did attend the face-to-face eczema education as part of their usual care at the hospital, only 1 in 5 families actually received the eczema education as intended. This means that many families were potentially under- or misinformed about effective eczema care strategies, with implications for the eczema care that they were providing to their children.

Finally, most children were recruited via the hospital outpatient clinic, and many had been attending the clinic for some time. It is possible that a subset of the children had refractory disease despite already receiving maximal therapy; however, the generally low volumes of topical treatments that were being used suggests this alone is unlikely to explain the lack of effect on disease or symptom severity or treatment use and adherence.

### Economic evaluation

Economic evaluation revealed a mean cost of AUD$159 per participating parent with parenting behavior (clinically) improved, and a cost of AUD$87 per participant for a scenario of ongoing full-capacity service delivery by trained practitioners. The cost per participant of delivering the intervention in the current trial was significantly impacted by the cost of training two new Triple P practitioners, particularly significant for a small cohort of participants.

The modeled costs for future intervention assume that trained practitioners are delivering the program. Without these training costs, and an increase in group capacity, the cost reduces to AUD$87 per participant from recurrent costs only. However, costs increase if existing or newly recruited staff are not already trained, although much lower than the AUD$320 per participant during the trial. The cost per participant would be further reduced if the newly trained staff were to be retained and be involved in delivering the program in future years, thus spreading the additional training costs over multiple years of program delivery. These results add to the literature on the costs versus benefits of delivering evidence-based parenting intervention to families (Sampaio et al., 2022) and supports the feasibility of delivering brief group-based parenting intervention to families of children with eczema in a pediatric setting via trained practitioners from within the healthcare team.

### Limitations

Difficulties with recruitment resulted in the trial being underpowered and only able to detect medium-to-large effects. Our recruitment rate (27.5%) was comparable to that from a previous trial with families of children with type 1 diabetes (22.7%), and similar issues (e.g., time, distance, caregiving responsibilities; Morawska et al., 2019) seem to have affected engagement in the current trial. Parenting interventions typically yield participation rates below 30%, and systematic reviews provide only limited evidence for strategies to increase recruitment, enrolment, or first attendance rates (Gonzalez et al., 2018). However, families in our trial appreciated the convenience of online delivery during the COVID-19 lockdown, and some expressed interest in a fully self-directed online format. Future research should explore parents’ perspectives on facilitators and barriers to participation, and whether flexible modes of delivery (e.g., self-directed online) could boost parent engagement in future trials. Second, most children in the study had only mild eczema, limiting our ability to detect improvements and potentially limiting the generalizability of results to clinically similar groups. Third, families self-selected into the study and tended to have higher levels of parent education and intact family structures compared to the broader population; what effect selection bias may have had on results is unknown. Finally, the economic evaluation is limited by the assumption that all groups are delivered at full capacity (12 participants/group), and cost per participant would increase if full capacity was not reached.

## Conclusion

*Healthy Living Triple P* is effective in reducing use of ineffective parenting practices and improving parents’ self-efficacy and task performance when managing children’s eczema and eczema-related behavior difficulties. These outcomes are important predictors of good eczema management and reduced disease severity. Further work is needed to establish whether regular symptom monitoring and provision of standardized eczema education moderates intervention effects, to reduce barriers to access, and to identify characteristics of children and families that predict better intervention update, engagement, and efficacy.

## Supplementary Material

jsae023_Supplementary_Data

## Notes

**Australian New Zealand Clinical Trials Registration:** ACTRN12618001332213
