# Supplementary material for: Brief parenting intervention (Triple P) for families of children with eczema: a randomized controlled trial
Source: J Pediatr Psychol. 2024 Apr 10;49(6):429–41. doi: 10.1093/jpepsy/jsae023 (PMC11175588; doi:10.1093/jpepsy/jsae023)
Supplement: jsae023_Supplementary_Data [file jsae023_supplementary_data.zip › jsae023_Supplementary_Data/jpepsy-2023-0167-File008.docx]

| 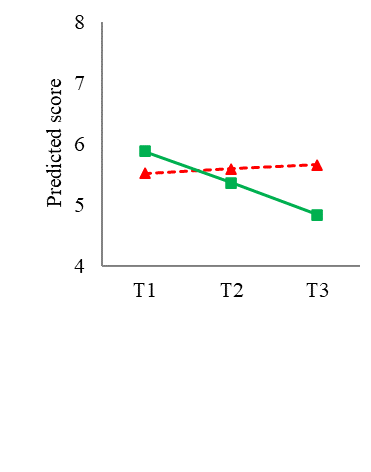 | 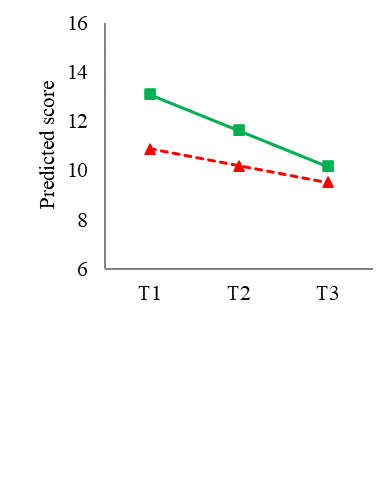 |  |
| --- | --- | --- |
| **a.** Clinician-rated eczema severity (*p*=.683) | **b.** Parent-report eczema severity (*p*=.394) |  |
|  |  |  |
| 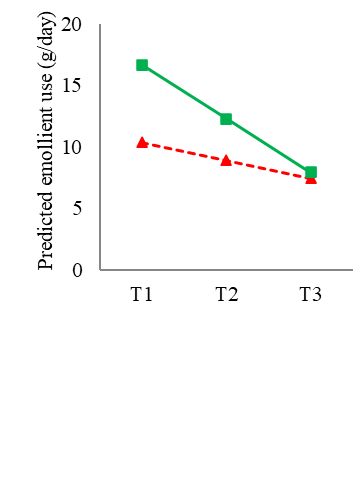 | 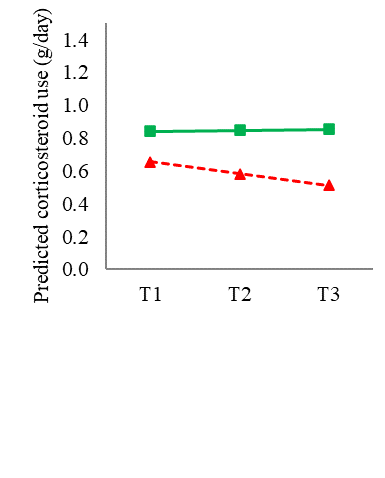 | 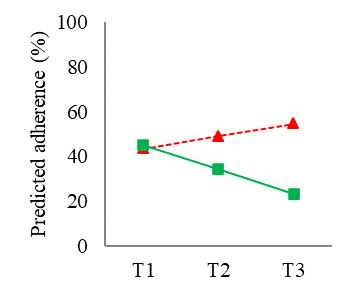 |
| **c.** Emollient use (p=.189) | **d.** Corticosteroid use (p=.650) | **e.** Corticosteroid adherence (p=.095) |
| **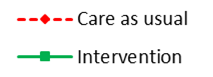** |  |  |
|  | | |
| **Supplementary Material - Figure S1.** Plots of regression slopes for change over time for primary outcomes (disease and symptom severity, treatment use) by treatment group. T1=baseline; T2=6-weeks post-intervention; T3=6-month follow-up. | | |

| 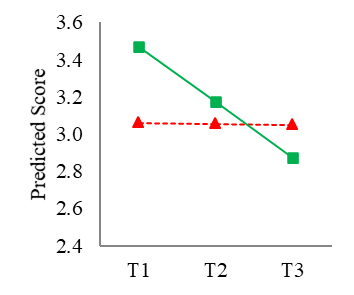 | 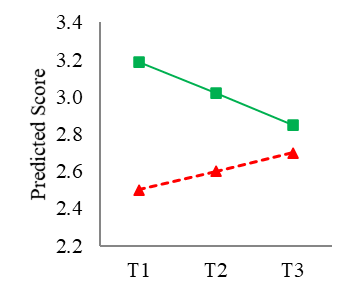 | 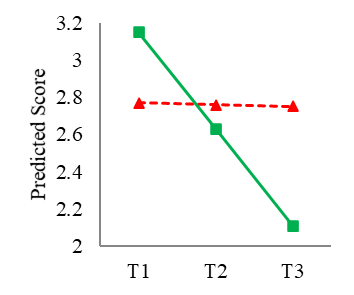 |
| --- | --- | --- |
| **a.** Ineffective parenting (*p*=.002) | **b.** Overreactivity (*p*=.018) | **c.** Laxness (*p*<.001) |
|  |  |  |
| 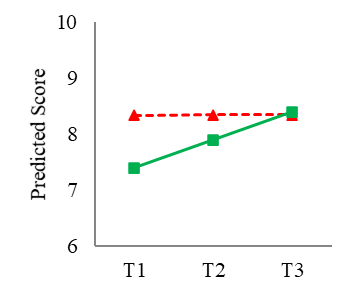 | 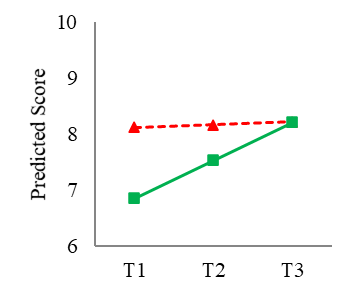 | 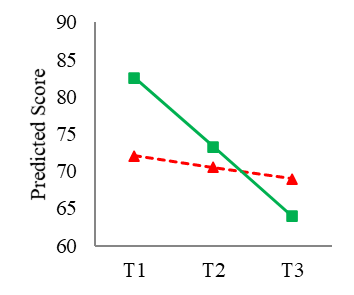 |
| **d.** Eczema management self-efficacy (*p*=.008) | **e.** Eczema management task performance (*p*=.003) | **f.** Eczema behaviour extent (*p*=.048) |
|  |  |  |
| 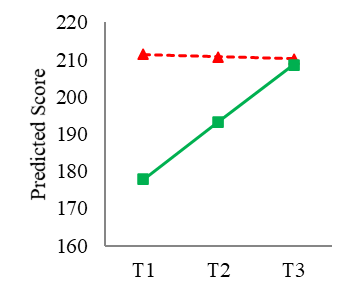 | 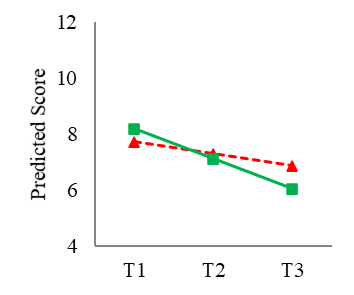 | 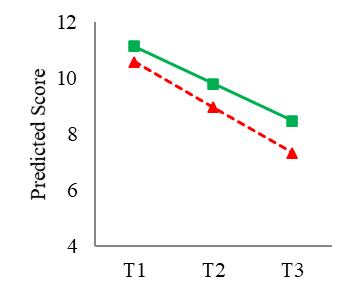 |
| **g.** Eczema behaviour confidence (*p*=.003) | **h.** Impact on child QoL (*p*=.371) | **i.** Impact on family QoL (*p*=.775) |
|  |  |  |
| **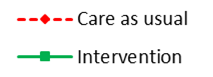** |  |  |
|  | | |
| **Supplementary Material - Figure S2.** Plots of regression slopes for change over time for secondary outcomes (psychosocial outcomes) by treatment group. T1=baseline; T2=6-weeks post-intervention; T3=6-month follow-up; QoL=quality of life. | | |

**Supplementary Material - Table S1.** Proportion of participants in Intervention and Care as Usual groups with clinically significant change on primary outcomes (disease severity and symptom severity) from T1-T2 and T1-T3

| Measure | Time | Condition | Clinically improved | | | Clinically worsened | | | No change |
| --- | --- | --- | --- | --- | --- | --- | --- | --- | --- |
|  |  |  | % (*n*/*n*) | χ^2 a^ | *p* | % (*n/n*) | χ^2 a^ | *p* | % (*n*/*n*) |
| Disease severity | T1-T2 | INT | 4.5 (1/22) | 0.23 | .629 | 13.6 (3/22) | 0.05 | .820 | 81.8 (18/22) |
|  |  | CAU | 8.0 (2/25) |  |  | 16.0 (4/25) |  |  | 76.0 (19/25) |
|  | T1-T3 | INT | 10.0 (2/20) | 0.07 | .795 | 5.0 (1/20) | 1.47 | .225 | 85.0 (17/20) |
|  |  | CAU | 12.5 (3/24) |  |  | 16.7 (4/24) |  |  | 70.8 (17/24) |
|  |  |  |  |  |  |  |  |  |  |
| Symptom severity | T1-T2 | INT | 34.8 (8/23) | 0.01 | .941 | 34.8 (8/23) | 0.04 | .846 | 30.4 (7/23) |
|  |  | CAU | 40.0 (10/25) |  |  | 28.0 (7/25) |  |  | 32.0 (8/25) |
|  | T1-T3 | INT | 42.9 (9/21) | 0.07 | .796 | 23.8 (5/21) | <0.01 | .982 | 33.3 (7/21) |
|  |  | CAU | 42.3 (11/26) |  |  | 19.2 (5/26) |  |  | 38.5 (10/26) |
| *Note.* T1=time 1 (baseline); T2=time 2 (post-intervention); T3=time 3 (6-month follow-up).  ^a^Pearson’s chi-square test for independence using Yates’ Continuity Correction with 1 degree of freedom, 2-tailed *p* value for Fishers’ Exact Test reported where expected frequency for any cell is <10. | | | | | | | | | |

**Supplementary Material - Table S2.** Proportion of participants in Intervention and Care as Usual groups with reliable and clinically significant change on secondary outcomes (psychosocial variables) from T1-T2 and T1-T3

| Measure | Time | Condition | Clinically improved | | | Reliably improved | | | Clinically and reliably improved | | | No change |
| --- | --- | --- | --- | --- | --- | --- | --- | --- | --- | --- | --- | --- |
|  |  |  | % (*n/*N) | χ^2^ ^a^ | *p* | % (*n*/N) | *χ*^2 a^ | *p* | % (*n/N*) | *χ*^2 a^ | *p* | % (*n*/*n*) |
| *Parenting practices* |  |  |  |  |  |  |  |  |  |  |  |  |
| Ineffective parenting | T1-T2 | INT | 53.8 (7/13) | 2.05 | .085 | 21.7 (5/23) | 3.96 | **.02** | 13.0 (3/23) | 1.61 | .10 | 78.3 (18/23) |
|  |  | CAU | 12.5 (1/8) |  |  | 0 (0/25) |  |  | 0 (0/25) |  |  | 96.0 (24/25) |
|  | T1-T3 | INT | 54.5 (6/11) | 4.10 | **.018** | 14.3 (3/21) | 1.94 | .08 | 14.3 (3/21) | 1.94 | .08 | 85.7 (18/21) |
|  |  | CAU | 0 (0/8) |  |  | 0 (0/26) |  |  | 0 (0/26) |  |  | 96.2 (25/26) |
| Laxness | T1-T2 | INT | 62.5 (5/8) | 0.63 | .315 | 13.0 (3/23) | 1.61 | .10 | 13.0 (3/23) | 1.61 | .10 | 87.0 (20/23) |
|  |  | CAU | 28.6 (2/7) |  |  | 0 (0/25) |  |  | 0 (0/25) |  |  | 96.0 (24/25) |
|  | T1-T3 | INT | 83.3 (5/6) | 0.85 | .266 | 19.0 (4/21) | 3.24 | **.03** | 14.3 (3/21) | 1.94 | .08 | 81.0 (17/21) |
|  |  | CAU | 42.9 (3/7) |  |  | 0 (0/26) |  |  | 0 (0/26) |  |  | 96.2 (25/26) |
| Overreactivity | T1-T2 | INT | 50.0 (6/12) | 0.73 | .316 | 13.0 (3/23) | 0.37 | .34 | 13.0 (3/23) | 0.37 | .34 | 87.0 (20/23) |
|  |  | CAU | 16.7 (1/6) |  |  | 3.6 (1/25) |  |  | 4.0 (1/25) |  |  | 96.0 (24/25) |
|  | T1-T3 | INT | 41.7 (5/12) | 1.29 | .245 | 9.5 (2/21) | 0.78 | .19 | 9.5 (2/21) | 0.78 | .19 | 90.5 (19/21) |
|  |  | CAU | 0 (0/5) |  |  | 0 (0/26) |  |  | 0 (0/26) |  |  | 96.2 (25/26) |
| *Note. Clinically improved* indicates movement of scores from the clinical to the non-clinical range based on published clinical cut-offs. *Reliably improved* indicates that scores have statistically reliably improved based on reliable change indices (Ineffective parenting=0.84 points, Laxness=1.31 points, Overreactivity=1.33 points; Jacobson & Truax, 1991). *Clinically and reliably improved* is the intersection of both. T1=time 1 (baseline); T2=time 2 (post-intervention); T3=time 3 (6-month follow-up). ^a^Pearson’s chi-square test for independence using Yates’ Continuity Correction with 1 degree of freedom, 2-tailed *p* value for Fishers’ Exact Test reported where expected frequency for any cell is <10. | | | | | | | | | | | | |

**Supplementary Material - Table S3.** Costs of delivering *Healthy Living Triple P* intervention during trial

| Cost Categories | Description | Cost per unit  (AUD 2019) | # Units | Total Cost   (AUD 2019) |
| --- | --- | --- | --- | --- |
| FIXED COSTS | | | | |
| Training costs | *Triple P practitioner training and accreditation (20 hour*s) | | | |
|  | Course fees | $1,255 per course | 2 courses | $ 2,510 |
|  | Practitioner @ $59.77/hr (plus 40% on costs) | $1,674 per practitioner | 2 practitioners | $ 3,347 |
| **Total fixed costs** |  |  |  | **$ 5,857** |
| RECURRENT COSTS | | | | |
| Session 1: | *Healthy Living Triple P – Session 1 (4 hours)* | | | |
|  | Practitioner @ $59.77/hr (plus 40% on costs) | $335 per session | 4 groups | $ 1,339 |
| Session 2: | *Healthy Living Triple P – Session 2 (4 hours)* | | | |
|  | Practitioner @ $59.77/hr (plus 40% on costs) | $335 per session | 4 groups | $ 1,339 |
| Capital | Seminar room @ QCH | $300 per day | 8 x half-days | $ 1,200 |
| Consumables | Teaching workbooks | $5.87 per workbook | 31 books | $ 182 |
| **Total recurrent costs** | |  |  | **$ 4,060** |
| **Total costs** | |  |  | **$ 9,917** |

*Note.* QCH=Queensland Children’s Hospital. The nominated cost of a seminar room at QCH was quoted at $300/day by the Finance Department.

**Supplementary Material - Table S4.** Impact of Triple P practitioner training and accreditation costs on cost per participant

| Period | Number of participants | Cost per participant (AUD 2019) | |
| --- | --- | --- | --- |
|  |  | Training costs included | Training costs excluded |
| Trial | 31 | $320 | $131 |
| Ongoing model |  |  |  |
| 100% capacity | 300 | $106 | $87 |
| 75% capacity | 225 | $114 | $87 |
| 50% capacity | 150 | $129 | $87 |
| *Note.* Training costs per participant assumes each group is delivered at full capacity. | | | |
